# Avian Malaria in Penguins: Diagnostics and Future Direction in the Context of Climate Change

**DOI:** 10.3390/ani12050600

**Published:** 2022-02-28

**Authors:** Kate Ings, Daniela Denk

**Affiliations:** 1 Garscube Campus, School of Veterinary Medicine, College of Medical, Veterinary and Life Sciences, University of Glasgow, Bearsden Road, Glasgow G61 1QH, UK; 2478157i@student.gla.ac.uk; 2 Institute of Veterinary Pathology, Centre for Clinical Veterinary Medicine, Ludwig-Maximilians-University, 80539 Munich, Germany

**Keywords:** avian, malaria, penguin, *Plasmodium*, diagnostics, PCR, climate change

## Abstract

**Simple Summary:**

Avian malaria is caused by infection with protozoa of the genus *Plasmodium*. This vector-borne parasite is spread by mosquitoes and has a variable significance depending on environmental, host, mosquito and parasite factors. Captive penguins in non-native environments are exposed to the protozoa without having coevolved with them and are especially sensitive to infection. The most common presentation of the disease in affected penguins is acute death. Infection of wild penguins is reported and a greater understanding of the significance of such infections is required. Global warming and related surges in vector availability present an increasing threat to conservation in captive environments and targeted research into the early diagnosis of disease is required. Current diagnostic methods predominantly rely upon direct microscopy and/or molecular testing on tissues obtained from penguin postmortem examinations, and frequently fail to identify the causative agent at a species level. There are several barriers to the development of a rapid method to detect infection and the causative species; however, this information would further our understanding of this disease, and development of such a method is a valuable undertaking. This paper provides a summary of current diagnostic methods, identifies the likely future impacts of avian malaria in penguins, and highlights the need to improve both the speed and scope of available diagnostics.

**Abstract:**

Avian malaria is caused by infection with haemoprotozoa of the genus *Plasmodium*. Infection is endemic in large parts of the world and is typically subclinical in birds that are native to these regions. Several penguin species have evolved in non-endemic regions without the selective pressure that these parasites exert and are highly susceptible to infection when transplanted to endemic regions, for example, in the context of zoological collections or rehabilitation centers. Avian malaria in penguins typically causes acute mortality without premonitory signs, or less commonly, nonspecific signs of morbidity, followed by mortality. Additionally, infection is reported in wild penguins, though the significance of these infections remains equivocal. As global temperatures continue to increase, avian malaria is likely to pose a continued and further threat to conservation efforts in captive environments. Intra vitam diagnosis currently relies on the evaluation of blood smears and molecular methods. The former is unreliable in penguins, as the acute clinical course typically does not allow the development of parasitemia. This absence of parasitemia also makes speciation challenging. Current molecular methods typically target the Cytochrome B or 18s subunit and have proven variably sensitive and specific. Reliable intra vitam diagnosis of avian malaria and further information about the causative agents at a species level would be very valuable in understanding the epidemiology and likely future course of avian malaria infection in penguins, and in particular, the implications avian malaria may have for conservation efforts. This paper provides an overview of malaria in penguins, discusses its changing impact on management and conservation, offers a summary of current diagnostics, and suggests future direction for the development of diagnostic tests. The latter will be key in understanding and managing this disease.

## 1. Introduction

Avian malaria is a highly significant disease of captive and rehabilitating penguins, caused by infection with intracellular protozoan parasites of the genus *Plasmodium*. *Plasmodium* (Plasmodiidae), along with the genera *Leukocytozoon* (Leukocytozoidae) and *Haemoproteus* (Haemoprotidae), are the most widely studied members of the order Haemosporidia [1,2,3,4]. Historically, little distinction between these genera was implied by the term ‘avian malaria’; however, in more recent years, it has been used exclusively to describe infections with parasites of the genus *Plasmodium* [2]. Approximately half of avian species have been examined for *Plasmodium* species infection and the vast majority of orders are affected [2]. Over 50 species of *Plasmodium* parasites have been observed to infect birds so far [4]. These can be divided into five subgenera: *Haemamoeba*, *Giovannolaia*, *Novyella*, *Bennettinia* and *Huffia* [2]. Avian-infective *Plasmodium* parasites are considered extremely generalist parasites and as such, their host range can be very large. For example, *Plasmodium relictum* is reported to infect over 70 avian families and the severity of infection can vary markedly depending on the host species, ranging from minimal or imperceptible reductions in health or fitness, as in the case of native passerines, to the rapid mortality reported in penguins [1,2,4,5,6].

## 2. Avian Malaria in Penguins

In general, avian malaria causes minimal impact on the fitness and survival of its avian hosts; however, a minority of groups of birds are especially sensitive to infection, chief amongst which is the family Spheniscidae [1,2,7,8,9]. *Plasmodium* species, which belong to the subgenera *Haemamoeba* (particularly *P. relictum)* and *Huffia* (particularly *P. elongatum*), seem to be the most pathogenic to penguins, and the mortality in captive and rehabilitating penguins ranges between 50 and 80% [1,7]. In addition, rare infections with *P. cathemerium*, *P. juxtanucleare*, *P. tejerai*, *P. nucleuphilum,* and *P. unalis* are reported [1,7]. There are a number of factors that influence the *Plasmodium* species of concern. Geographical distribution and abundance are particularly important. For example, *P. relictum* predominantly affects penguins housed in Europe, whilst both *P. relictum* and *P. elongatum* are commonly reported in penguins captive in North America [7]. This is thought to primarily reflect the parasitic species present in native wild bird populations, rather than a distinct sensitivity of the local penguins to these species [7]. Infections with avian malaria have been reported in the Northern (*Eudyptes moseleyi*) and Southern (*Eudyptes chrysocome*) Rockhopper, King (*Aptenodytes patagonicus*), Humboldt (*Spheniscus humboldti*), African (*Spheniscus demersus*), Magellanic (*Spheniscus magellanicus*), Snares (*Eudyptes robustus*), Little (*Eudyptula minor*), Yellow eyed (*Megadyptes antipodes*), Chinstrap (*Pygoscelis antarcticus*), Macaroni (*Eudyptes chrysolophus*), and Gentoo (*Pygocelis papua*) penguins [5,6,10,11,12,13]. Humboldt (*Spheniscus humboldti*) and African (*Spheniscus demersus*) penguins seem to be the most frequently affected, although this likely reflects a bias towards keeping those species in captivity [10]. The species in which infection is not reported typically do not encounter the vector, as they are native to regions inhospitable to mosquitos (Antarctica in particular) and have either not been kept in captivity or are maintained in mosquito-proof captive environments. Thus, their lack of disease is most likely attributable to a lack of exposure rather than a resistance to infection [5]. The list of *Plasmodium* species causing infection in penguins is unlikely to be exhaustive for several reasons: challenges in accessing cases of mortality in isolated wild colonies, in addition to the challenges inherent in speciating avian malaria parasites in minimally parasitemic birds; the presence of as yet undefined species (for example unidentified *Plasmodium* species have been observed in penguins in Brazil [7]); and the incomplete and frequently overlapping keys used for identification. A number of publications have sought to clarify these areas, however work is ongoing [4,11].

The rates of avian malaria infection of wild Spheniscidae vary with penguin species, geography, and the detection method used. Infection is less common in wild birds when compared with captive birds in endemic habitats, likely related to the immunosuppressive circumstances surrounding captivity (e.g., morbidity leading to rehabilitation, stress, movement, etc.), as well as the relative freedom of movement and environmental conditions (particularly wind) experienced by free-range penguins [10]. Despite this, infections with parasites of the genus *Plasmodium* have been reported in several wild penguin colonies (Table 1). A number of studies have highlighted a freedom from infection in some wild colonies. For example, 501 Humboldt and 360 Magellanic penguins sampled from across South America were negative following PCR testing [12]. Although the authors speculated that latent infections, which typically occur within the viscera, may not be detected in blood samples [12]. Environmental stressors are also suspected to play a role in wild penguin susceptibility; for example, 31% of African penguins from South Africa were found to be seropositive for *Plasmodium* species infection, which rose to 55% in oiled birds [13].

## 3. Clinical Disease

Clinical signs of avian malaria infection in penguins typically range from minimal to absent prior to acute death; although, less commonly, signs such as pale mucous membrane, lethargy, anorexia, vomiting, dyspnea and/or behavioral separation are reported [1,2]. Occasional reports of neurological manifestations, including incoordination, seizure and paralysis, are attributed to the obstruction of cerebral microvasculature following endothelial expansion by developing meronts. Additionally, endothelial hypoxia itself induces thrombosis and further cerebral anoxia [1,2,17]. Acute death may occur secondary to circulatory shock following the release of large numbers of inflammatory mediators (cytokine storm), to respiratory insufficiency, or to cardiac tamponade due to hydropericardium [1,2]. Hmatocrit is often used in mammalian infections to monitor disease. In birds, packed cell volume reductions of up to 50% are recognized, however, the reliability of this in penguins is likely to be minimal, as mortality typically occurs prior to significant parasitemia and the resulting erythrocyte rupture [2]. The very acute clinical course of disease in penguins and the absence of widely available rapid diagnostic tests make treatment very challenging or frequently impossible.

The long-term impact of chronic infection with *Plasmodium* parasites is not currently fully understood. Due to the typically acute nature of the disease in penguins there is a marked absence of information in this area [10]. Therefore, our understanding is based on work performed in other avian species. Here, growing evidence suggests that, despite minimal short-term behavioral alterations, chronic avian malaria infection significantly reduces longevity and fitness, including brood size, chick quality, and fledging success in a variety of avian species [17,18,19]. Underlying reasons for this likely include reduced cellular survival, metabolic demand, and increased free radical generation [17,18,19,20,21]. Furthermore, a study by Delhaye et al. demonstrated that latent *Plasmodium* species infections are liable to recrudesce on exposure to a novel pathogen, indicating that not only is there an ongoing immunosuppression of the *Plasmodium* species by the host, but that the resources devoted to mounting an immune response are finite and a portion is devoted to suppression of the parasites in chronic cases [17]. A similar process of energy allocation is likely to underlie the observation that there are higher malarial burdens in birds with large brood sizes [22]. The true cost of this immune response is difficult to distinguish from the metabolic effect of the presence of the haemoparasites [22]. In conclusion, a significant body of evidence suggests long-term reductions in fitness and survival in cases of chronic malaria; however, the extent to which this is applicable to penguins, where chronic disease is less commonly documented, remains unclear.

## 4. Epidemiology

Work investigating *Plasmodium gallinaceum* in the 1940s remains the basis for our understanding of the life cycle of *Plasmodium* species [4,23,24]. Overall, the life cycle can be summarized as follows [1,2]: Sporozoites in the saliva of female vectors are injected into susceptible birds. They undergo asexual reproduction (merogony) within fibroblasts or macrophages local to the bite, forming cryptozoites. These cryptozoites release meronts, which infect the lymphohistiocytic cells of the viscera and undergo a second cycle of merogony. These secondary merozoites have three possible fates: they may infect endothelial cells (with the exception of members of the subgenus *Huffia*, which do not infect endothelial cells) and become exoerythrocytic meronts; they may reinfect solid tissues and become phanerozoites; or they may infect erythrocytes. Depending on their host cell type, they will either undergo additional cycles of asexual merogony (all host cells) or form gametocytes (erythrocytes only). Gametocytes remain within circulation and are infectious to biting insects at the next blood meal. Once within the midgut of the vector they undergo gametogenesis, which results in the formation of macrogametes and microgametes. These form a motile ookinete which penetrates the gut wall and begins development of an oocyst. This oocyst undergoes sporogony and ultimately ruptures, releasing vast numbers of sporozoites into the haemocoel of the vector. Sporozoites migrate to the salivary ducts within the haemocoel. Variation in the intricacies of this process between *Plasmodium* species is expected, given the wide range of avian hosts, vectors, geographic regions, and parasite species implicated in avian malarial infections. Indeed, avian-infective *Plasmodium* parasites are more variable in both their host and vector specificity when compared with their mammalian-infecting counterparts. Avian-infective parasites of the genus *Plasmodium* have been reported to infect birds across different orders, whilst vector specificity remains poorly understood for a wide range of *Plasmodium* species [3,4,25]. For those which have been better studied, a wide range of dipteran species and even genera have been demonstrated to support sporogony [3,4,25,26]. A greater understanding of species-specific life cycles is likely to significantly enhance the understanding and treatment of avian malaria infections.

Infected captive penguins are frequently unable to survive parasitemia and therefore cannot sustain an infection. In such cases, local avifauna are the proposed reservoir of infection [1,2]. In these native host birds, the peak in parasitemia (known as the ‘crisis’) occurs within 6–12 days after the first appearance of parasites in the blood [2]. The parasite levels in the blood decrease markedly following development of an immune response; however, the parasite may persist for the lifetime of the bird and can recrudescent in times of stress [1,2]. Additionally, there is evidence that some passerine hosts can remain infective for prolonged periods (up to 1 year) and therefore represent reservoirs of infection. In addition, their relative tolerance for infection may also facilitate the evolution of more virulent *Plasmodium* species [1,2,27]. This local reservoir of infection makes eradication and prevention of disease in captive birds highly challenging [10,28].

Susceptibility of penguins to malarial infection is significantly associated with a number of physiological and environmental factors including molt, chick rearing, husbandry problems, concurrent disease, etc. [1]. This is attributed to catecholamine-mediated immunosuppression [6]. A range of concurrent disease processes are reported in penguins with malaria, including aspergillosis, enteritis, septicemia, chlamydiosis, Avipoxvirus and West Nile virus infection, various helminths, amyloidosis, and cholestasis. Whether malarial birds are more susceptible to secondary disease or the various primary disease processes render birds more susceptible to malaria remains equivocal [1]. Immunologically naïve penguins are particularly at risk of malarial infection and as such, outbreaks of avian malaria in captive penguin colonies are typically associated with birds in exotic/zoological collections that have not co-evolved with the parasite [6,15]. In endemic countries, infection (including in non-native resident birds) is often seasonal, with an increased number of birds affected in late spring and early summer, a phenomenon known as the “spring relapse” [6]. This increase in infectious pressure occurs for several reasons: an increasing photoperiod causing increased circulating corticosterone levels resulting in immunosuppression; an increase in the total avian population due to migration; and an increased vector population as the climate becomes warmer [6,18].

## 5. Monitoring, Prevention and Treatment

Rising global temperatures are anticipated to increase the threat that avian malaria infection poses to penguins. A survey of over 3000 avian species revealed an upward trend in infections with *Plasmodium* species over the past 20 years, attributed to alterations of the lifecycle and range of the mosquito vector due to increasing global temperatures [23,24,25,26,27]. These higher temperatures are anticipated to increase both vector range and abundance, the latter through increased availability of suitable sites for larval maturation, longer breeding seasons, and a reduced interval between blood meals and oogenesis [23,24,25,26,29]. Interestingly, a link between ambient temperature and parasitemia is not well evidenced, possibly because the latter also reflects the host immune response [27]. This increased vector activity is likely to lead to the exposure of previously naïve avifauna, including a number of endangered species, and could have devastating effects [23,24]. In addition, host specificity of *Plasmodium* parasites is reported to vary with climatic conditions; regions with more pronounced seasonality of rainfall and with higher rainfall in dry periods are associated with less generalist *Plasmodium* species [25]. This is attributed to fluctuating resource availably leading to a clustering of hosts, particularly adjacent to water supplies, an increased seasonality of host behavior which synchronizes them spatiotemporally and physiologically, and altered parasitic transmission and development [25]. The implications of these findings in the context of climate change remain equivocal.

Several penguin species are considered by the International Union for Conservation of Nature to be endangered or at risk, amongst these are the inhabitants of malaria-adjacent regions [30,31,32,33,34,35,36,37,38,39,40,41,42]. Extension of the range of the mosquito vector into these regions may lead to the exposure and endangerment of previously naïve penguin populations. At present, active surveillance is ongoing in birds from the Galapagos Islands and New Zealand. In both cases, the fragile avifauna and introduction of *Culex quinquefasciatus* are particular causes for concern [15,43,44]. Furthermore, infection in Antarctica is not yet reported and the possible impact of the mosquito vector’s range reaching to the southern extremes of the planet are considerable and potentially devastating [15,43,44].

In addition to the monitoring of at-risk penguin populations, a range of malarial prophylactic measures have been proposed. In both captive and wild scenarios, mosquito control measures have been suggested, such as the introduction of infertile or malaria-resistant mosquitoes, and the alteration of the environment to reduce the possibility of larval development [33]. Housed birds also benefit from mosquito-proof enclosures and chemoprophylaxis, although the implementation and maintenance of such regimens can be challenging [3,45,46]. The development of human malarial vaccines is an area of great interest. Recently the RTS, S/AS01E vaccine has demonstrated positive effects on the clinical malaria risk in children (albeit with an intensive vaccination regimen) [47]. DNA vaccines for avian malaria trialed in canaries and captive penguins also proved protective for the first year [9]. However, vaccinated birds were highly susceptible to malaria the subsequent year, likely due to a failure of the development of a protective immune response [2,8,9].

In conclusion, there is no single effective protective measure currently available for wild birds. Captive birds may be successfully managed with a combination of approaches, but these regimens are challenging to establish and implement on a long-term basis, and success rates vary greatly amongst collections [48].

## 6. Current Diagnostics

A range of approaches are currently utilized in the diagnosis of avian malaria. Specific challenges are recognized in the case of penguins, predominately relating to the acute course of disease and minimal parasitemia. As a result, most diagnoses of *Plasmodium* infection in captive penguins occur post mortem. Gross postmortem lesions can be highly suggestive, but are not considered pathognomonic [1]. The most common findings include splenomegaly, hepatomegaly, pulmonary oedema, hydropericardium, pneumonia, and/or myocarditis in animals in good body condition [1].

### 6.1. Examination of Blood Smears

Identification of intraerythrocytic meronts by direct microscopy of Giemsa-stained blood smears has been the traditional mean of diagnosing avian malarial infection—intraerythrocytic merogony is not seen in other Haemosporidia and is therefore pathognomonic [1,2]. As mentioned previously, this method is significantly less reliable in penguins, which frequently fail to demonstrate blood stages in the course of acute disease. Chronic infection in avian species is typically pauciparasitic and therefore also challenging to diagnose on smears. This may explain the relative scarcity of reported *Plasmodium* infections when compared to other Haemosporidia [1,2]. Further challenges to the diagnosis of avian malaria by blood smear assessment include operator factors (skill levels and experience), the quality of the blood smear and stain, and the number and life stage of the parasitic species present [4]. An updated key to avian malaria parasites was published by Valkiunas & Iezhova in 2018, detailing 55 *Plasmodium* morphospecies. The authors consider this key incomplete and have identified a number of possible species which require further investigation [4].

### 6.2. Post-Mortem Cytology, Histopathology and In Situ Hybridization

**Figure 1:**
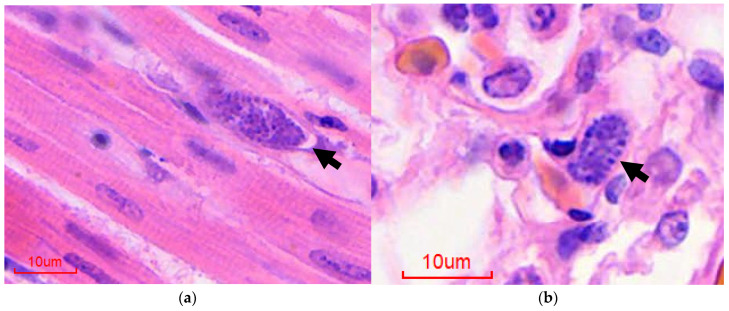
Histological diagnosis of avian malaria in penguins, hematoxylin and eosin-stained sections: (**a**) Heart, extraerythrocytic meront (arrow); (**b**) Lung, intraendothelial extraerythrocytic meront (arrow).

Rapid postmortem diagnosis can be achieved by detection of extraerythrocytic meronts on impression smears from the liver, spleen, heart, and lungs, obtained via direct impression smears at postmortem examination [1]. Histopathological evaluation of postmortem tissue is also a useful diagnostic tool; typical findings include granulocytic interstitial pneumonia, myocarditis and splenitis, and mononuclear hepatitis with intralesional protozoa (Figure 1) [1]. In addition, splenic hemosiderosis and extramedullary hematopoiesis are common features and the deposition of “malaria pigment” (birefringent hemozoin granules that are the iron-containing product of the parasitic breakdown of hemoglobin) in splenic and hepatic parenchyma is highly suggestive [1,2]. The presence of parasitic meronts in tissue is highly informative, though speciation based on published keys is challenging, depending on the nature of the tissue and the parasitic life stages present [3,4]. In addition, diagnosis based on tissues which have extensive postmortem artifacts or marked karyorrhexis is particularly challenging based on light microcopy alone, and detection relies heavily on the experience of the examining pathologist [12]. Furthermore, the increasing use of digital slides is felt to increase the difficulty of diagnosis in the authors’ experience [12]. In such cases, in-situ hybridization (ISH), which has been validated for use in formalin fixed paraffin embedded (FFPE) tissues, may be employed [12]. This probe targets the small subunit, which was chosen because of its high copy number and therefore, its relative abundance within the parasite genome [49]. Unfortunately, the test is not currently commercially available [43]. The major drawback of visceral cytology, histopathology, and ISH is their reliance on tissues collected at postmortem examination. In addition, species-specific probes are not available, and therefore none of these methods can reliably identify *Plasmodium* infection at a species level [12,44,49].

### 6.3. Enzyme-Linked Immunoassay (ELISA)

Serological assessment of both wild and captive penguins has been the subject of several studies, utilizing both human (*Plasmodium falciparum*) and avian (*Plasmodium relictum*) malarial antigens, to estimate the level of *Plasmodium* species challenge [13,45,46,50]. Overall, seropositivity is higher in captive or rehabilitating penguins than in their wild counterparts, and as would be expected, varies markedly amongst wild penguins based on geographical location [13,46]. For example, Graczyk et al. demonstrated 100% seropositivity in penguins from New Zealand, and 0% in Antarctic Adélie penguins, with a range of other species in various locations ranging between 33 and 92% [13]. ELISA-based testing methods are valuable in assessing exposure at a population level; in observing infectious trends over population, time, and geography; and are sensitive in diagnosing chronic disease. However, the typically acute nature of fatal disease in penguins does not allow sufficient time for the bird to generate a humoral response and therefore renders ELISA testing less useful in the context of an acute disease outbreak [12,51]. Even in more chronic disease, ELISA testing is unlikely to be useful as an individual prognostic tool. Graczyk et al. suggest that the antibody titer is not predictive of severity of disease or parasitemia [45]. In addition, maternal antibodies have been demonstrated to persist for between three and ten weeks, and ELISA testing is unlikely to be reliable in very young birds [23,45]. Therefore, the use of ELISA testing can be useful if assessing the exposure but is less likely to be applicable in the case of individual bird diagnosis or treatment.

### 6.4. Alternative Assays

In human medicine, a range of parasite markers have been targeted in the development of rapid diagnostic tests, including histidine-rich protein 2, aldolase, and lactate dehydrogenase (LDH) [52,53]. These metabolic markers are particularly useful, as they specifically identify live parasites [54]. Early results of one such human-focused test used on postmortem penguin blood samples appeared promising; however, additional research is required to evaluate the true diagnostic merit of such an approach [54]. More recently, a single report of a validated LDH-based method of detection was published; however, the test was reported to be no longer in use [10]. Novel targets for rapid bird-side testing may prove a significant area of research in the future.

### 6.5. Polymerase Chain Reaction

Polymerase chain reaction (PCR) is more sensitive for diagnosing avian malaria than light microscopic diagnosis. Indeed, studies performed on wild birds in American Samoa reported an incidence of 1% by microscopy, compared with almost 60% when tested by PCR [2,51]. A range of PCR tests have been developed for avian malaria, which typically target either the small subunit DNA (SSU-rDNA or 18S-rDNA), or the mitochondrial cytochrome-B gene (Cyt-B), although a range of other targets have been trialed [15,53,55,56,57,58,59]. Recently, those tests targeting the Cyt-B gene have proven more reliable and are now most commonly utilized. Indeed, since 2004, nested PCR targeting the Cyt-B subunit has been the most commonly published method of detecting avian malaria (61.7% of publications) [55,56,57,58,59]. The work on the Cyt-B lineages of avian malaria parasites has been significantly enhanced by the development of the MalAvi database, which provides a standardized reference of sequences by species, as well as detailing the phylogenetic markers, recorded host species and geographical range [11]. Apart from its use in research, PCR testing for avian malaria is not currently widely commercially available.

## 7. Diagnostic Challenges, Conclusion and Future Direction for Investigation

Despite their ubiquity in current research, there are several challenges inherent in the development of PCR tests for avian malaria. Some of these are intrinsic to the parasite. Avian-infecting *Plasmodium* species are known to have some of the most adenine (A) and thymine (T) rich genomes ever sequenced. These AT rich genomes are relatively homogenous, less stable than those with a greater guanine–cytosine (GC) content, and typically have a lower primer binding affinity, owing to the lesser strength of the AT vs the GC bonds formed during primer annealing [59,60]. As a result, these AT rich genomes present fewer suitable targets for primers and force selection of more AT rich sequences within target regions. AT rich primers require lower annealing and extension temperatures, which due to the nature of PCR reactions, leads to a greater risk of non-specific binding [61]. Additionally, the Haemosporidia are closely related, and current published PCR tests do not distinguish between species or genera without additional sequencing of the PCR product. Furthermore, traditional PCR methods frequently fail to identify mixed infections, as sequencing of the cloned product is not routinely preformed. Other studies report inadvertent co-amplification of multiple genera [62,63]. However more recent work by Ciloglu et al. demonstrates successful diagnosis of single and mixed haemosporidian infection at a genus level [64]. Multiplex PCR-ligase detection reaction assays have been utilized in human medicine to identify causative *Plasmodium* by species, but such work has not, to the authors’ knowledge, been carried out in avian malaria [65].

A sensitive PCR may, in theory, risk amplification of parasite DNA from non-competent hosts if an infected vector has recently injected parasitic DNA. This may be amplified in the absence of a true infection [56]. This is, however, considered unlikely to present a significant problem in practice, owing to the relative paucity of sporozoites.

Methods employed in an attempt to combat these challenges and improve PCR sensitivity include the use of nested PCR tests, first described for diagnosis of human infections in 1993 [66]. The principle of a nested PCR test is that two separate rounds of PCR are undertaken. The first round is performed with a set of primers, known as the outer primers, which flank and amplify a region of the genome. The second round is performed on the product of the first and employs a pair of primers which bind sequences within the product of the first reaction. The result is more sensitive and specific targeting of the region of interest, which is particularly valuable when the volume of target DNA is small and associated with a large volume of non-target genetic material [11,63,66,67,68].

Finally, the increased use of molecular methods, including sequencing of PCR products to identify *Plasmodium* species, is beginning to reveal the abundance of total species. This is true in particular for the amount of cryptic diversity which exists within the *Plasmodium* species, demonstrated by the frequently poor correlation between morphospecies and the outcome of molecular investigations [57,58]. Of the morphospecies identified in the most recent key, very few have been successfully identified using molecular markers; therefore, targeting probes/primers to identify species is an ongoing challenge [4].

## 8. Conclusions

In summary, avian malaria is a highly important disease affecting captive and rehabilitant penguins. Infection is associated with acute mortality and ante-mortem diagnosis is highly challenging. Rising global temperatures are associated with an increased pressure of *Plasmodium* species infection and an increasing host range. Therefore, it is feasible to assume an associated greater disease pressure in penguins. There are several challenges inherent in the diagnosis of avian malaria in penguins due to the acute clinical course of disease. In addition, several features of the parasite’s physiology and ecology make characterization of species and therefore species-specific diagnosis highly complex. The recent development of molecular methods has provided the basis for reliable diagnostics at a genus level and, in combination with genetic sequencing or direct microscopy, may be useful in species-level diagnosis. Despite this, there is an acute need for more definitive identification of infective species to allow for the investigation of the epidemiology of this highly important pathogen of penguins. Novel diagnostics using monoclonal antibodies targeting parasitic markers may be of value in the development of bird-side tests. Given the anticipated increase in the malarial exposure of Spheniscidae with increasing global temperatures, diagnosis and appropriate management will likely be critical in the conservation of these popular and iconic birds.

## Figures and Tables

**Table 1:** Reports of malaria in free ranging penguins.

Penguin Species	Location	Means of Diagnosis	*Plasmodium* Species	Reference
Black-footed *Spheniscus demersus*	South Africa	Blood smear	*P. relictum*	Brossy, 1992 [14]
	South Africa	Serology	-	Graczyk et al., 1995 [13]
Yellow eyed *Megadyptes antipodes*	New Zealand	Blood smear	*P. relictum*	Fantham and Porter, 1944 [6]
Snares *Eudylypes robustus*	New Zealand	Blood smear	*P. relictum*	Fantham and Porter, 1944 [6]
Northern rockhopper *Eudyptes moseleyi*	Gough Islands	Blood smear	*P. relictum*	Fantham and Porter, 1944 [6]
Galapagos *Spheniscus mendiculus*	Galapagos Islands	PCR	-	Levin et al., 2009 [15]
Little *Eudylyptes minor*	New Zealand	PCR	-	Jansen van Rensburg, 2010 [16]

References [6,13,14,15,16].

## Data Availability

No new data were created or analyzed in this study. Data sharing is not applicable to this article.
